# Stem Cell Therapy for Lower Extremity Diabetic Ulcers: Where Do We Stand?

**DOI:** 10.1155/2013/462179

**Published:** 2013-03-18

**Authors:** Mei Yang, Lingling Sheng, Tian R. Zhang, Qingfeng Li

**Affiliations:** ^1^Division of Plastic Surgery, Southern Illinois University School of Medicine, Springfield, IL 62794, USA; ^2^Department of Plastic and Reconstructive Surgery, Shanghai Ninth People's Hospital, Shanghai Jiao Tong University School of Medicine, Shanghai 200011, China

## Abstract

The impairment of wound healing in diabetic patients is an important clinical problem affecting millions of patients worldwide. Various clinical and basic science studies show that stem cell therapy, as a regenerative medical therapy, can be a good solution. In this paper, we begin with an introduction of the cellular mechanism of the diabetic ulcer. We will then discuss the advantages and limitations of various stem cell therapies that have been under extensive recent study.

## 1. Introduction 

 The diabetic ulcer, a major complication of diabetes mellitus, has remained an important clinical challenge. Not only does it affect the physical and mental health of patients, but it is also an economic burden on our society. Now, the diagnosis and classification of diabetic ulcer is guided by the Wagner classification system and the University of Texas Diabetic Wound Classification system [1]. The standard clinical treatment of diabetic ulcer includes local wound care with dressing, virgous and repeated debridement of necrotic tissue, and offloading. Antibiotics will also be given if infection exists. However, the result is still far from satisfaction, and 14%–20% of patients with diabetic ulcer will end up with amputation [1, 2].

Various approaches have been developed for diabetic wound healing, but most of these approaches have centered on one facet of wound healing, such as inflammation or growth factors [3–5]. With a multifactorial etiology, therapy that focuses on one facet has limited therapeutic efficacy [6, 7]. However, stem cell therapy has shown promise. Clinical and basic science studies show that these therapies can provide a comprehensive solution by addressing multiple factors during diabetic wound healing, including cell proliferation, extracellular matrix (ECM) synthesis, growth factor release, and vascularization. In this paper, we begin with an introduction of the cellular mechanism of the diabetic ulcer. We will then discuss the strengths and limitations of various stem cell therapies that have been under extensive recent study.

## 2. Diabetic Wound Healing

 Wound healing is a highly dynamic and complex process. When skin is injured, organic debris must be removed from the site of injury, and new components must be delivered or recreated there. These orchestrated processes involve mutual interactions between cells, extracellular matrix (ECM), and soluble factors [8, 9]. Neovascularization is also a crucial step in this process [9]. However, diabetic wound healing shows impaired cellular activity, ECM synthesis, growth factor release, neovascularization, and so forth, resulting in ulcer formation [10–17]. Factors that contribute to delayed wound healing in diabetic patients are discussed as follows. Some other factors, such as infection and oxidative stress, however, will not be discussed either because they are less related to stem cell therapy or because seldom any publication is available to address them. 

## 3. Impaired Cellular Activity

 Many cell lineages are involved in wound healing. Forty-eight hours after injury, keratinocytes migrate from stratum basale and differentiate to form new skin [18]. Usui et al. evaluated proliferation, differentiation, adhesion, and migration of keratinocytes along the margin of chronic ulcers from patients with diabetes mellitus. Compared with a normal wound healing, keratinocytes from the diabetic ulcer exhibited increased proliferation, decreased differentiation, and decreased migration ability. They concluded that the decreased migratory ability of keratinocytes from diabetic patients may contribute to the slow wound healing process [18]. Fibroblasts contribute to wound healing by synthesizing collagen to increase the strength of the wound [19]. However, in the diabetic wound, fibroblasts are found to have several abnormal characteristics, including less proliferation and excessive apoptosis [19, 20]. These diabetic fibroblasts also have impaired migration ability. It has been found that fibroblasts from diabetic mice migrate 75% less than those from normoglycemic mice. In addition, these epidermal cells have defective responses to growth factors and hypoxia, a condition commonly present in chronic wounds, which may also contribute to delayed wound healing in diabetic patients [10, 11, 21]. 

 Aside from these defective differentiated cells, abnormal stem cells also play a role in stalling wound healing. In a study of rat diabetic wound, Zhong et al. found that epidermal stem cells isolated from diabetic wounds showed decreased clone formation ability. By further examining the expression of beta-catenin and cyclin D1, these stem cells were found to be defective in the differentiation ability [12]. Adipose-derived stem cells (ADSCs), the stem cell in subcutaneous tissue, have also been studied. Differing from the aforementioned cells, ADSCs from both diabetic wounds and normal skin secreted identical amounts of growth factors, cytokines, and type I collagen. Diabetic ADSCs even proliferate at the same rate as those from normal wound healing [13]. These characteristics of ADSCs from diabetic wounds render them a potential cell source for wound healing and regeneration. 

## 4. ECM Synthesis

 ECM, composed of collagen, cell-adhesive glycoproteins, glycosaminoglycans, and proteoglycans, helps support cells and comprise key components of the basement membrane that anchors and helps replenish epidermal cells in normal skin. During wound healing, extracellular matrix components are active in each phase, interacting with cells and growth factors in a dynamic give and take that eventually results in wound closure [14]. Not only does it provide a scaffold for wound healing and regeneration, but also components of the ECM play key roles in stimulating cell proliferation and differentiation, guiding cell migration, and modulating cellular responses [8, 9]. The diabetic wound is characterized by deficient ECM synthesis and excessive ECM degradation. Deficient ECM synthesis is attributed to impaired fibroblast activity [22]. The excessive ECM degradation is a result of increased matrix metalloproteinase (MMP) production [20]. Deficiency of collagen, the main component of ECM, results in chronic connective tissue defects and impaired wound healing [15, 23]. 

## 5. Growth Factor Release

 During wound healing, growth factors released from platelets, macrophages, neutrophils, fibroblasts, keratinocytes, and endothelial cells influence every phase of wound healing by providing signals for various cellular activities. Epidermal growth factor (EGF), platelet-derived growth factor (PDGF), transforming growth factor-beta (TGF-beta), insulin-like growth factor 1 (IGF-1) vascular endothelial growth factor (VEGF), and fibroblast growth factor (FGF), are under intensive study. They are found to be closely related to epidermal cell proliferation, and some of them have been found to play an important role in angiogenesis by modulating endothelial cell migration and proliferation [24–28]. The diabetic wound is characterized by a reduced expression of nearly all of these growth factors [16]. 

## 6. Angiogenesis and Neovascularization

Angiogenesis and neovascularization are also an important part of wound healing. Angiogenesis refers to the sprouting of microvessels through a preexisting capillary network, whereas neovascularization refers to vascular formation from endothelial progenitor cells that differentiate or endothelial cells that proliferate *in situ* [29]. Not only do they provide nutrition and oxygen for wound healing, but also they bring inflammatory cells and circulating stem cells into the wound site. However, microcirculation damage is observed in diabetic wound healing [17, 30]. Multiple factors are involved in the damage of vascular supply. Recent studies show that damaged microcirculation in the diabetic wound is caused by endothelial dysfunction [17, 30]. Other studies suggest that low levels of VEGF as well as senesce in endothelial progenitor cells (EPCs) may also play a role in decreased local neovascularization during diabetic wound healing [31]. 

## 7. Inflammation

 Inflammation is a necessary part of normal wound healing, playing an important role in fighting infection, clearing debris, and inducing cell proliferation [8]. However, if prolonged, it can also lead to extra tissue damage, a phenomenon observed in diabetic wounds [32]. Studies show that prolonged inflammation in diabetic wounds is characterized by sustained expression of proinflammatory cytokines, such as interleukin-1 (IL-1) and TNF-alpha, and large numbers of polymorphonuclear neutrophils and macrophages, which delay epidermidalization [32]. Moreover, prolonged inflammation also leads to increased levels of MMPs, a family of proteases that can degrade the extracellular matrix proteins. During normal wound healing, various members of the MMP family are upregulated, and each of them cleaves a specific subset of matrix proteins. For example, MMP-9 can degrade proteins in the basal lamina and release keratinocytes to contribute to wound healing. However, misregulated expression of MMPs is found to contribute to impaired wound healing [8, 9]. In a clinical study of diabetic wound, by comparing the patients who achieved complete wound healing and those who developed diabetic ulcers, the authors found that both increased inflammation and expression of MMPs, especially MMP-9, are the main factors that contribute to diabetic ulcer formation [33]. Ibuki et al. found that the skin is fragile in diabetic mice, which is caused by the increased levels of oxidative stress and MMPs [34]. Moreover, the abnormal expression of MMPs also contributes to the ineffectiveness of exogenous growth factor therapy [33–36]. 

## 8. Stem Cell Therapy

 Stem cells are considered the master cells, capable of both self-renewal and multilineage differentiation [37]. Stem cell therapy, which refers to an interventional strategy that introduces adult stem cells into damaged tissue in order to treat disease or injury, has been studied as a treatment for diabetic ulcers [37]. Because of ethical problems associated with embryonic stem cells, we will focus on adult stem cell therapies, including mesenchymal stem cells (MSCs), endothelial progenitor cells (EPCs), bone-marrow-derived mononuclear cells (BM-MNCs), and fibrocytes (Table 1). 

## 9. Mesenchymal Stem Cells

 Mesenchymal stem cells (MSCs) are multipotent stromal cells that were first found in bone marrow by Friedenstein et al. in 1966 [38]. MSCs were later found in various other tissue types, including bone marrow, umbilical cord blood, adipose tissue, and amniotic membrane. There is no consensus on the characterization of these mesenchymal stem cells. The criterion proposed by the Mesenchymal and Tissue Stem Cell Committee of the International Society for Cellular Therapy is commonly used in research. According to this criterion, MSCs must be plastic adherent when maintained in standard culture conditions. MSCs must express CD105, CD73, and CD90 and lack expression of CD45, CD34, CD14 or CD11b, CD79alpha or CD19, and HLA-DR surface molecules. MSCs must be able to differentiate into osteoblasts, adipocytes, and chondroblasts *in vitro* [39]. Mesenchymal stem cells are the most common stem cell in clinical and basic science studies. Their multidirectional differentiation, easy collection, and weak immunogenicity make them a good source for therapy [40]. Various studies show that MSC transplantation can have multiple effects on diabetic wound healing, including promoting cell proliferation, collagen synthesis, growth factor release, wound contraction, neovascularization, and cellular recruitment to wounds [41]. 

 Bone-marrow-derived mesenchymal stem cells (BM-MSCs), also known as marrow stromal cells, are fibroblast-like self-renewing stem cells in the bone marrow [40]. The BM-MSCs are nearly 10% of the hematopoietic stem cells (HSCs) in number, and they are always regarded as a component of the HSC niche [37]. 

 In a rat diabetic wound healing model, Kwon et al. found that systemic and local treatment with BM-MSCs on diabetic wounds improved the breaking strength by increasing collagen levels (types I–V) in the wound bed [42]. Higher expression of various growth factors has also been observed in numerous studies, including EGF, KGF, TGF-beta1, VEGF, SDGF-1alpha, IGF-1, IL-8, PDGF, and angiopoietin-1 [18–21, 42]. These growth factors contribute to the repair, the regeneration, and the neovascularization in the diabetic wound. One interesting finding about these growth factors is that in addition to their role in vascularization *in situ*, they can also recruit stem cells from the circulation or even from bone marrow to participate in angiogenesis [43]. Now, growing evidence shows that paracrine secretion of growth factors is the main therapeutic mechanism of these stem cells [28]. Another possible mechanism may be their direct transdifferentiation into vascular endothelial cells and skin components [44–46]. However, due to the low engraftment after transplantation and lower rate of differentiation *in vivo*, this mechanism is considered less important than the paracrine secretion effect [33, 41]. 

 ADSCs have been referred to as processed lipoaspirate cells (PLA), adipose-derived stromal cells, and adipose-derived mesenchymal progenitor cells. The nomenclature differences also reflect a lack of consensus and an evolving knowledge of the phenotype and function of these cells. Although the phenotype characterization of ADSCs generally follows the criteria proposed by the International Society for Cellular Therapy, some disputes still exist, especially the question that whether ADSCs are CD34 positive or negative [47, 48]. Both CD34+ and CD34− ADSCs have been considered as ADSCs [49]. Several recent studies show that CD34+ ADSCs have a greater proliferative capacity, while CD34− ADSCs have a greater differentiating capacity [47, 49, 50]. 

 Kim et al. studied the effect of human adipose-derived stem cells on healing of ischemic wounds in diabetic nude mice. The authors found that ADSC-treated animals had an earlier and abundant neovessel formation and better tissue remodeling than the control group without any treatment. Lower rates of autoamputation and a survival rate comparable to group II were also observed in the ADSC-treated group [51]. In an *in vitro* study, Lee et al. used human ADSC culture medium to treat human keratinocytes and dermal fibroblasts. The authors found increased cell proliferation in both cell types. Moreover, the expression of collagen I was also increased [47]. 

 MSCs have been applied clinically for the treatment of diabetic wounds. Despite significant beneficial results, an *in vitro* expansion time is still in need to acquire a sufficient amount of cells for treatment. Long *in vitro* expansion time and multiple handling procedures are barriers for its clinical application and increase chances of infection [52]. 

## 10. Endothelial Progenitor Cells

EPCs were first isolated from peripheral blood and are characterized by expression of CD34, KDR (VEGFR-2), and CD133 markers. Later, EPCs were also found in bone marrow and umbilical cord blood [29]. 

There are studies showing that EPCs can be recruited from bone marrow or peripheral blood to the sites of neovascularization to participate in various normal and pathological processes, including wound healing and ischemic injury [53]. Studies have also been performed to evaluate therapeutic potential of EPCs in diabetic wound healing. In a mouse wound healing model, Lee at al. [54] found that EPCs can promote wound healing and neovascularization. In their study, embryonic-stem-cell- (hESC-) derived EPCs were used for wound healing. Rapid reformation of granulation tissue and reepithelialization of wounds were observed after treatment. By further exploration of therapeutic mechanism, they found higher expression of EGF, bFGF, fractalkine, granulocyte-macrophage colony-stimulating factor (GM-CSF), interleukin (IL)-6, IL-8, platelet-derived growth factor-AA (PDGF-AA), and VEGF. They proposed that the therapeutic mechanism might be the secretion of growth factors and cytokines [54]. Besides their paracrine secretory effect, EPCs also have potential to differentiate into endothelial cells. Moreover, in some studies of ischemic injury, EPCs were found to migrate to the ischemic area from peripheral blood and even bone marrow [29, 55]. The clinical application of EPCs also experiences similar problem as MSCs [52].

## 11. Bone-Marrow-Derived Mononuclear Cells

BM-MNCs are a group cells composed of many kinds of stem cells and differentiated cells, including hematopoietic stem cells, mesenchymal stem cells, endothelial progenitor cells, precursor cells, and their progeny [56]. MNCs are abundant in both peripheral blood and bone marrow, and they can be collected directly for transplantation without *in vitro* expansion [56]. 

As complex cell fractions, BM-MNCs have multiple cell markers. The most important cell populations involved in angiogenesis are hematopoietic progenitor cells (CD133+, CD117+, and CD34+) and MSCs, which are CD34 negative. Among MSCs, the endothelial progenitor population, composed chiefly of CD34−/CD133+/VEGFR2+ and CD34+/CD133+/VEGFR2+ cells, plays the most important role in vascular regeneration process [57]. 

 In a diabetic mouse wound healing model, Sivan-Loukianova et al. found that during a five-day observation period, treatment with MNCs could accelerate epidermal healing and rapidly and dramatically accelerate revascularization of the wounds. During the initial treatment period, increased vascularization was mediated principally by an increase in vessel diameter. Later, both an increase in vessel size and number were observed [58]. There are also some clinical studies about MNC therapy: Ruiz-Salmeron et al. performed autologous MNC transplantation in diabetic patients with peripheral artery disease. After 3 to 12 months, all patients exhibited clinical improvement with a significant increase in vascular network [59]. Similar improvements have been observed in other clinical trials [60, 61]. However, the therapeutic mechanism remains unclear. Some studies show that the elevated expression of angiogenic growth factors is observed after MNC transplantation. Other studies suggest that MNC transplantation can decrease local inflammation [45]. One study shows that a small portion of MNCs may differentiate into endothelial cells, indicating direct contribution to neovascularization [56, 62, 63]. The complex makeup of MNCs makes it difficult to further elucidate the therapeutic mechanism. Despite this, the avoidance of *in vitro* expansion makes MNCs practical for clinical application. 

## 12. Fibrocytes

 The “fibrocyte” was described in 1994 as a circulating, bone-marrow-derived cell with the ability to adopt a mesenchymal phenotype [64]. Fibrocytes are mostly found in peripheral blood, and they comprise 0.1% to 0.5% of the total leukocyte population [65]. They exhibit spindle-shaped morphology when cultured *in vitro*. This cell bears features of both fibroblasts and monocytes, and this combination of connective tissue cell and myeloid features allows its identification by a number of markers, such as CD34, CD11b+, CD13+, MHC II+, CD86+, and CD45+. They are also positive for some stromal cell markers such as collagen I, vimentin, and fibronectin [65, 66]. Some studies show fibrocytes promote wound healing by increasing ECM deposition, wound contraction, and vascularization [67]. After local injection of fibrocytes, Kao et al. found that these cells can accelerate wound healing by stimulating cell proliferation, reepithelialization, and angiogenesis [67]. Higher expression of growth factors (VEGF, b-FGF, TGF-beta, PDGF-A, and FGF-7), chemokines (MCP-1 and MIP-1alpha), and extracellular matrix (collagen I and alpha-SMA) was observed in fibrocyte-treated wounds, suggesting that the paracrine secretion effect of fibrocytes might contribute to diabetic wound healing [65, 67, 68]. The *in vivo* differentiation of fibrocytes has remained unclear. Some studies suggest that fibrocytes may contribute to wound healing by differentiating into fibroblasts and myoblasts [69]. However, other studies suggest that fibrocytes are mesenchymal progenitors and can differentiate into mesenchymal stem cells *in vivo* [70]. Further studies are needed to explore the therapeutic potential of these cells.

## 13. Conclusions

 The diabetic ulcer remains an important clinical challenge in the current medical practice, and much effort has been focused on the development of novel therapeutic approaches for its treatment. Various stem cell therapies have shown promise, and pertinent clinical trials are summarized in Table 2. Stem cell transplantation can provide systemic improvement to the wound site, including enhancement in cell proliferation, ECM synthesis, growth factor release, and neovascularization. Transplanted stem cells can act as a “biological pump” to secrete various growth factors. They can also contribute to wound healing by differentiating into skin or vascular components. Despite their great therapeutic potential, some questions still remain. First, most of these cells, except MNCs, need *in vitro* expansion to acquire sufficient numbers, greatly limiting clinical application. Second, most stem cell therapies, for clinical use, are achieved through autologous transplantation. That brings up the issue that whether stem cells isolated from diabetic patients are still “normal” and whether there is some method to reprogram them to “normal.” Some research shows that diabetic EPCs are functionally impaired, possibly requiring as of yet undescribed methods to restore them to normal [79, 82]. Finally, there is still concern that these stem cells may not be oncologically safe as some studies show that stem cells may contribute to tumor growth or even become the source of tumors [83, 84]. Further study is needed to answer these questions and to make stem cell therapies more practical in the clinical setting. 

## Figures and Tables

**Table 1:** Stem cells and their therapeutic effects.

Cell type	Cell markers	Therapeutic effect
BM-MSCs	CD105+, CD73+, CD90+, CD45−, CD34−, CD14−, CD11b−, CD79 alpha, CD19−, and HLA-DR− [37–39]	Promote cell proliferation, collagen synthesis, growth factor release, wound contraction, neovascularization, and cellular recruitment to wounds [41–46].
ADSCs	CD31−, CD34+/−, CD45−, CD90+, CD105−, and CD146− [47–50]	Increase cell proliferation, collagen synthesis, promote neovessel formation, and tissue remodeling [47, 51, 52].
EPCs	CD34+, VEGFR-2+, and CD133+ [29, 40]	Promote vascularization secrete proangiogenic growth factors and cytokines, and differentiate into endothelial cells [53–55].
BMNCs	hematopoietic progenitor cell markers: CD133+, CD117+, and CD34 MSCs markers and endothelial progenitor population: CD34+/−, CD133+, and VEGFR2+ [56, 57]	Secrete angiogenic growth factors decrease local inflammation, and promote vascularization differentiate into endothelial cells [58–63].
Fibrocytes	CD 34+, CD11b+, CD13+, MHC II+, CD86+, CD45+, collagen-1+, procollagen-1+, CD3−, CD4−, CD8−, CD19−, and CD25− [64, 65]	Increasing cell proliferation ECM deposition, wound contraction, and vascularization. Secrete of growth factors and chemokines [65–70].

**Table 2:** Clinical trials of stem cell therapy on the treatment of diabetic ulcer.

Clinical trial	Diabetic cases	Stem cell therapy	Efficacy assessment
Kirana et al. 2012 [71]	24	BM-MSCs/BM-MNCs	80% cases achieved significant improvement.
Ravari et al. 2011 [60]	8	BM-MNCs	Complete wound healing in 3 cases and significant improvement in 5 cases.
Jain et al. 2011 [72]	25	BM-MNCs	The rate of chronic lower extremity wound healing increased.
Dash et al. 2009 [73]	12	BM-MSCs	Significant improvement in pain-free walking distance and reduction in ulcer size.
Rogers et al. 2008 [74]	3	BM-MNCs	Good wound healing.
Yoshikawa et al. 2008 [75]	20	MSCs	The wound mostly healed in 18 of the 20 patients.
Kirana et al. 2007 [61]	1	BM-MNCs	Complete wound healing achieved.
Badiavas et al. 2007 [76]	4	BM-MNCs	Complete wound healing achieved.
Falanga et al. 2007 [77]	8	MSCs	All wounds healed completely between weeks 7 and 8 after the surgery.
Asai et al. 2006 [78]	1	BM-MNCs and bFGF	Complete wound healing.
Humpert et al. 2005 [79]	2	BM-MNCs	Reduction of wound size and markedly increased vascularization were achieved.
Vojtassák et al. 2006 [80]	1	Fibroblast and MSCs	Closing and healing of the nonhealing diabetic ulcer was achieved.
Badiavas and Falanga 2003 [81]	3	BM-MNCs	Dermal rebuilding and closure of nonhealing chronic wounds were achieved.

## Abbreviations

ECM: Extracellular matrix
ADSCs: Adipose-derived stem cells
MMP: Matrix metallo proteinase
EGF: Epidermal growth factor
PDGF: Platelet-derived growth factor
TGF-beta: Transforming growth factor-beta
IGF-1: Insulin-like growth factor 1
VEGF: Vascular endothelial growth factor
FGF: Fibroblast growth factor
KGF: Keratinocyte growth factor
SDGF-1alpha: Stromal cell-derived factor-1 alpha
IL-8: Interleukin-8
GM-CSF: Granulocyte-macrophage colony-stimulating factor
EPCs: Endothelial progenitor cells
MSCs: Mesenchymal stem cells
BM-MNCs: Bone-marrow-derived mononuclear cells
MCP-1: Monocyte chemotactic protein-1
MIPs: Macrophage inflammatory proteins.
